# Operational Variables on the Processing of Porous Titanium Bodies by Gelation of Slurries with an Expansive Porogen

**DOI:** 10.3390/ma14164744

**Published:** 2021-08-22

**Authors:** Antonio Javier Sanchez-Herencia, Zoilo Gonzalez, Alejandro Rodriguez, Esther Molero, Begoña Ferrari

**Affiliations:** 1 Institute for Ceramic and Glass (ICV-CSIC), Kelsen, 5, 28049 Madrid, Spain; q42gogrz@uco.es (Z.G.); or emolero@icv.csic.es (E.M.); bferrari@icv.csic.es (B.F.); 2 Chemical Engineering Department, Faculty of Science, Campus de Rabanales, Universidad de Cordoba, Building Marie-Curie, 14071 Cordoba, Spain; a.rodriguez@uco.es; 3 Department of Mechanical Engineering, University of Cordoba, Medina Azahara Avenue, 14071 Cordoba, Spain

**Keywords:** colloidal processing, porosity, gelation, porogen, experimental design, compression resistance

## Abstract

Colloidal processing techniques, based on the suspension of powders in a liquid, are very versatile techniques to fabricate porous structures. They can provide customized pores, shapes and surfaces through the control of operational parameters, being the base of the alternative additive manufacture processes. In this work disperse and stable titanium aqueous slurries has been formulated in order to process porous materials by the incorporation of methylcellulose (MC) as a gelation agent and ammonium bicarbonate as an expansive porogen. After casting the slurries and heating at mild temperatures (60–80 °C) the methylcellulose gels and traps the gas bubbles generated by the ammonium bicarbonate decomposition to finally obtain stiff porous green structures. Using an experimental design method, the influence of the temperature as well as the concentration of gelation agent and porogen on the viscosity, apparent density and pore size distribution is analyzed by a second-order polynomial function in order to identifying the influence of the operating variables in the green titanium porous compact. After sintering at 1100 °C under high vacuum, titanium sponges with 39% of open porosity and almost no close porosity were obtained.

## 1. Introduction

Titanium and titanium-based materials are widely used for many applications, including in the aerospace and terrestrial industries, because of the unique combination of excellent mechanical properties, low density and corrosion resistance in most aggressive environments [1]. Metallic titanium and its alloys are expensive materials to produce from ingots and fabrication from powder-metallurgical techniques offers an effective means to reduce the cost of titanium parts as it produces near-net shapes, thus minimizing material waste.

Additionally, to the excellent mechanical and chemical properties, titanium and its alloys are biocompatible, what makes this family of materials the most widely employed for bone prosthesis and replacements. Despite these good properties to be used as structural bone replacement, to acquire effective fixation of the metal to the natural bone the porosity became a critical factor regarding to the mismatch of Young’s moduli between biomaterial and the surrounding bone [2]. Porosity is also relevant for different biological processes related to the tissue growth and angiogenesis during the osseointegration of the implants, i.e., pore sizes lower than 1 µm are responsible of the interactions with proteins; inside porosities ranging between 1 µm and 20 µm are where the interactions with cells occur. Finally, connected porosities ranging between 100 µm and 1000 µm are necessaries for vasculogenesis processes [3,4].

A porous material has to be considered a composite where the pores are one of the phases characterized by their size, shape, amount and distribution and the connectivity through walls is an important characteristic, since holes in the pore walls should create a continuous voids structure that reach the surface of the pieces. The ability to process open cell foams results in a significant extension of values of important properties, such as the density, conductivity, the Young’s modulus and the compressive strength [5]. In fact, porous titanium can be found playing a key role in many industrial applications, including filtration, separation, catalyst supporting, gas absorbing, gas sparging, current collection and the mentioned medical implantation [6].

**Figure 1:**
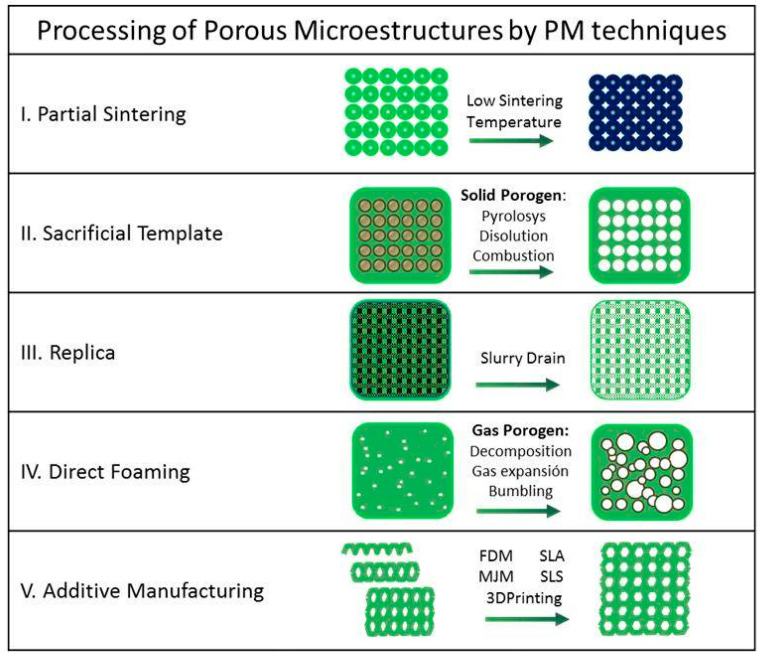
Scheme of Powder Metallurgy techniques for porous metal processing.

Processing strategies for porous metallic compacts intend to mimic the three porosity levels to facilitate the mechanical and osteointegrative responses at all morphological stages [2,5,7]. The well-known powder metallurgical (PM) strategies to process porous titanium (and titanium-based materials) mainly consist on partial sintering of green pieces; the mixture of dry powders with space-holder or sacrificial templates that afterwards decomposes or dissolves; the direct foaming or the additive manufacturing techniques such as selective laser melting or electron beam melting (Figure 1) [8]. Moreover, the increasing relevance of the macroscopic shape of the implants [9]-in addition to the composition and the pore structure- as well as the topological design of their surface [10] demands an extensive control on the fabrication methods. In this melt a new cohort of techniques arises such as the rapid prototyping or the near net shaping methods based on the colloidal processing technologies. These techniques can provide customized pores, shapes and surfaces through the control of operational parameters and the formulation of the slurries or inks.

Colloidal processing techniques, based on the suspension of powders in a liquid, have proved to be versatile techniques to fabricate complex structures [11]. The use of liquid media for powders mixing as well as available processing additives such as binder, plasticizers, thermoplastic, gels, etc., open new opportunities for the design of porous structures. In addition, colloidal methods are the base of the alternative additive manufacture techniques which use printable inks and slurries, such as tape casting, direct writing, inkjet printing, robocasting or even fusion deposition modeling [12].

The use of suspension of metallic powders on water or other liquid to process compacts or coatings has gained special attention in the last two decades for two main reasons. The first one is that it will extend to metallic materials the use of well-known and developed ceramics processing techniques, such as spray-drying [13,14], slip casting [15] or gel casting providing some economic and clean processing alternatives for compacts with complex microstructures [16]. The second one is that complex compositions of metals and metal-ceramics powders and compacts, with an intimate mixture of micrometric and nanosized phases, can be better achieved in liquid media by dispersing and stabilizing micro and nanoparticles with different morphologies and high shape factors. Some of these methods has been successfully used to shape porous structures using Ti slurries or pastes, by slip casting [17,18], gel casting [19], foaming with H_2_O_2_ [20], freeze-casting [21], replication [22] or robocasting [23]. 

In the colloidal techniques, the contents of stabilizers and processing additives are critical formulation parameters defining the microstructural characteristics, and then on the final response of porous structures [24,25]. The complexity of the slurry formulation and the increasing number of processing parameters makes the experimental design a necessary tool to analyze the influence of each factor in the final result. In the present work a shaping route based on a liquid-solid-gas colloidal system was proposed for the formation of macroporous structures. In this specific case the use of ammonium bicarbonate is proposed, which decomposes within the gelation temperature range of a gelation agent as the methylcellulose (MC), releasing NH_3_ and CO_2_ according to the equation:

(NH_4_) HCO_3_ → H_2_O + NH_3_ + CO_2_ (1)

By simultaneous gelation and decomposition of the ammonium bicarbonate, the released gases will be trapped in the gel network. Consequently, the ammonium bicarbonate will take the role of the pore former or porogen, while the role of the MC is not restricted to the powder compaction with a defined shape since it will also trap the CO_2_ bubbles leading to a porous Ti structure. The weight of the different processing variables in the characteristics of the green Ti sponge was analyzed by an experimental design method, and the sintered properties of the Ti open sponge cell were determined. The methodology presented in this paper provides processing tools to design green porous bodies with variables sizes and porosity by controlling the interlaced initial composition and processing temperature.

## 2. Materials and Methods

### 2.1. Slurry Formulation and Characterization

Starting powders where commercial metallic Ti fabricated by plasma spray under inert atmosphere (AP&C Inc. Boisbriand, QC, Canada) with a mean particle size of 10 µm. As pore former ammonium bicarbonate of analytical grade (Panreac, Barcelona, Spain) was used. As dispersing agent, a polyethylenimine (PEI) with a molecular weight of 2000 g/mol (Aldrich, Schnelldorf, Germany) was used, while as gelling additive a low viscosity (4000 mPa·s) methylcellulose A4M grade supplied by Dow Chemicals (Midland, MI, USA) from. The pH was fixed with tetramethylammonium hydroxide.

Aqueous suspensions of as-received Ti powders were prepared by mixing the particles in DI water containing the proper amount of dispersant at a pH of 9 under mechanical stirring. The slurry was later ball milled in a plastic jar with balls for 2 h. Suspensions for gelation experiments were prepared by dissolving the methylcellulose into deionized water to a further addition of the dispersant in the required amount, and a pH shifting to 9 with TMAH. Over this basic solution, the pore former was dissolved to later on add the Ti powder, and ball mill in the same conditions and time used for the plain Ti slurries. Suspensions for thermal gelation and pores generation were prepared dissolving the ammonium bicarbonate with MC in the liquid medium of the slurry and following a similar protocol that this described previously.

Rheological measurements of concentrated slurries were recorded using a rheometer (Haake Mars II, Karlsruhe, Germany) and a double cone-plate measuring geometry. Tests were performed working on control rate (CR) and control stress (CS) modes. For CR tests, shear rate was increased up to 1000 s^−1^ in 120 s, keep at 1000 s^−1^ for 60 s and decreased to 0 s^−1^ in 120 s. In the case of CS mode tests, the stress was increased from 0 to the desired value and back to 0 Pa at up and down rate of 2 Pa/min. The applied high-shear rates during up-ramps are enough to achieve a reproducible suspension microstructure, dependent only on the suspension composition, but not on the slurry preparation history [16]. Then the flow curves presented are the down-ramps in the log–log plot and fitting following the Cross model (1):

(2) $$\eta =\eta_{\infty }+\left[\frac{\eta_{0}-\eta_{\infty }}{1+{\left(C\right)}^{n}}\right]$$

where *η*_0_ and *η*_∞_ are the extrapolation of the viscosity to zero (zero-shear viscosity) and infinity (infinite shear viscosity), respectively, *C* is a time constant and n is the rate constant, and it is a parameter which is related with the dependence of viscosity on the shear rate. The Cross model describes the limit behavior of the standing suspension and at an infinite shear rate, therefore not only gives information about which suspension is more viscous but which is more stable.

### 2.2. Ti Sponges Processing and Characterization

Porous compacts were fabricated by pouring the slurries into flexible silicone molds and heating them in a chamber with forced convection at temperatures ranging between 60 and 80 °C until all the water was evaporated. During heating, gelation and drying processes take place. The porous green samples were then cool down at room temperature and demolded. The characterization of the green bodies consisted in the measure of the pore size distributions by Mercury Intrusion Porosimetry (MIP) (Pore Sizer 9300, Quantachrome Instruments Co., Boynton Beach, FL, USA) while apparent densities were calculated geometrically using 5 samples for each heating temperature considering theoretical density of Ti as 4.51 g/cm^3^.

The green Ti samples were sintered in a vacuum furnace (Carbolite HVT 15/50/45, Neuhausen, Germany) at 10^−5^ atm at 1100 °C for 30 min. The characterization of sintered parts consisted in the measurement of their density at room temperature by the Archimedes method using an analytical balance with a resolution of ±0.1 mg, He pycnometry (MonosorbMultipycnometer, Quantachrome Instruments Co., Boynton Beach, FL, USA) and MIP. Compressive mechanical tests were carried on using a universal testing machine (Microtest S.A., Madrid, Spain) with steel plates. The pressure was applied using a load frame displacement rate of 0.02 mm/min. Load and displacement of the load frame were recorded during pressing. The microstructure of the sintered samples was registered by a scanning electron microscope (Philips Model XL30, Eindhoven, The Netherlands). The oxygen content, which is an interstitial element and has the greatest effect on the mechanical properties of Ti was measured using an inert gas fusion technique. For these measurements, a LECO TC-500 (MI, USA) was used.

### 2.3. Experimental Design Method

To obtain equations that allow estimating the quality of materials from the process variables, a factorial design was additionally applied, which allows the development of an empirical model to propose optimal operating conditions. To quantify the effects of these operational variables on the dependent variables, a 2^n^ factorial design was used. This experimental design works with a series of points (experiments) around a point of central composition (central experiment), for the estimation of constant for the mathematical models tested. This design satisfies the general requirements and therefore all parameters of the mathematical models can be estimated without an excessive number of experiments. The design used is defined by three parameters: number of variables, k; constant p, which takes the values 0 for k < 5 and 1 for k > 5; and number of central points, nc.

These parameters originate from three groups of points:

- 2*^k−p^* points of the factorial design.
- 2·*k* axial points.
- *n_c_* central points.

The number of total points, *n* (experiments) is calculated by the equation:

(3) $$n=2^{k-p}+2\cdot k+n_{c}$$

Considering that we are working with three operational processing variables, *n* = 15 (The variables combination required to construct the model is the result of the 8 points of the factorial design, 6 axial points and 1 central point). 

The operational variables were normalized by using the following expression:

(4) $$X_{n}=2\frac{\left(X-X_{m}\right)}{\left(X_{max}-X_{min}\right)}$$

where *X_n_* is the normalized value of gelation agent (X_G_), porogen (X_p_) or temperature (X_T_); *X* is the absolute experimental value of the variable concerned; *X_m_* is the mean of the extreme values of *X*; and *X_max_* and *X_min_* are its maximum and minimum values, respectively. 

The normalized values of the operational variables used in this test for the 15 experiments are included as supplementary material (Table S1). They were labelled in each experiment with values of −1, 0 and 1. X_G_ is the concentration of MC and takes values of 8, 10 and 12 g/L, X_P_ is the concentration of ammonium bicarbonate and takes values of 15, 20 and 25 wt.% related to the solid content of the slurry and X_T_ is gelation/drying temperature and takes values of 60, 70 and 80 °C.

## 3. Results

### 3.1. Study of the Influence of the Operation Variables on the Properties of the Ti Sponges

Ti slurries were formulated by incorporating ammonium bicarbonate as the porogen agent and MC as the shaping additive. Dispersion of the Ti powders in water has been previously reported by authors in terms of zeta potential and rheological behavior using an anionic and a cationic dispersant as well as bare powders [26,27]. On those papers, the Krieger Dougherty’s rheological model showed that using PEI (cationic dispersant) in the slurries formulation the values of maximum packing density were as high as 74% while the use of an anionic polyelectrolyte (Polyacrylic Acid-PAA, Mw = 2000, Acros) generated a maximum packing density value of 56%. Additionally, the Ti suspensions with solid content of 50 vol.% prepared with 1 wt.% of PEI as dispersant enabled the use of different shaping technologies. For that reason, in this paper the same solid content (50 vol.%) as well as the same dispersant concentration (1 wt.% of PEI referred to powder) has been used to the rheological studies of the slurries with the gelation agent. 

Experimental design method consisting of a central test and 14 peripheral tests on the sides and vertices of a cube were used to study the influence of three different variables on the processing of Ti porous structures by gel casting from colloidal aqueous suspensions (with a solids content of 50 vol.% and 1 wt.% of PEI at pH 9). The aim of applying this statistical tool was to define the equations that predict the final properties of a similar material (dependent variables) from the parameters of the process (operating variables). Operating variables were the gelation agent concentrations (X_G_), the porogen concentrations (X_P_) and temperature (X_T_). 

**Figure 2:**
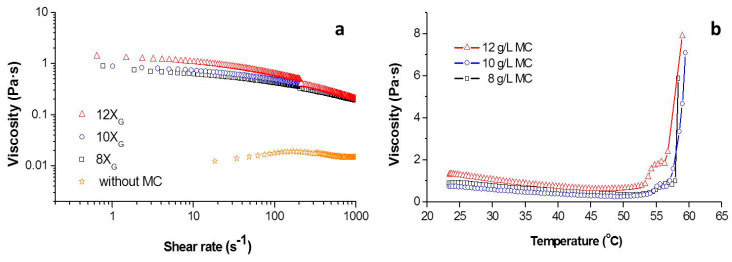
(**a**) Flow curves of Ti slurries with 50 vol. % of solid contents formulated without MC and with 8, 10 and 12 g/L of MC. (**b**) Viscosity values vs. Temperature for 50 vol.% slurries with 8, 10 and 12 g/L of MC.

The amount of gelation agent (X_G_ variable) determines the polymerization degree and the gel structure during the gelation process, being also the responsible of changes in the rheological behavior of the suspensions. For this reason, the addition of different amounts of MC to the Ti slurries was firstly studied in terms of its rheological behaviour. Methylcelluloses are common thickeners for liquids used in food industries, biotechnology and materials processing. Rheological modification depends on intrinsic characteristic properties such as the molecular weight and substitutions of the anhydroglucose repeat unit as well as concentration of additive. Amounts can range from a few grams per litter [28] to tens of grams [29]. Figure 2a shows the viscosity vs. the shear rate for slurries of Ti with 50 vol.% of solids contents in DI water (without gelation agent or porogen) and in an aqueous media containing 8, 10 and 12 g/L of MC (X_G_8, X_G_10 and X_G_12). For each of the slurries the curves were made overlapping the decreasing shear rate of the CR and CS experiments. It should be noted that for the thicker slurries the CR and CS curves do not coincide on the overlapping zone, as the CR experiment shears the slurry more energetically leaving no time for rearrangement of interparticle forces, while the CS experiment is less energetic allowing a faster formation of the mentioned interparticles forces and consequently higher viscosity values.

The decreasing on the viscosity values with the increment of shear rate indicated a pseudoplastic behavior of Ti + MC slurries. As expected, the addition of the gelation agent increased the viscosity, being this increment more significant at the low shear rates. In addition, the thickening effect of the initial Ti suspension disappeared as consequence of the MC addition. All experimental curves were fitted to the Cross model in order to obtain the values for the zero-shear viscosity (*η*_0_) and the infinite shear viscosity (*η*_∞_). High values of zero-shear viscosity are indicative of the capacity of samples to keep the shape and avoid segregation of phases in a resting state (out of external forces or stirring). On the other hand, low infinite shear viscosity values are indicative of efficiency on the mixing and milling processes. These values are collected in Table 1 together to the values of viscosity at shear rates of 10 and 100 s^−1^ which are reference values for casting. 

It can be clearly observed that under similar dispersion conditions (1 wt.% PEI) the media containing 12 g/L of MC presented a higher zero-shear viscosity, denoting a higher capability to keep the shape after the forming processes and consequently prevent the formation of closed porosity into the slurry. Furthermore, the media containing 8 g/L of MC resulted in values of *η*_∞_ below 100 mPa·s which indicates a higher homogenization capability. The viscosity values of all MC slurries in the shear range of 10–100 s^−1^ fulfilled the requirements to be processed by gel casting.

In Figure 2b the variation of the viscosity of the slurries recorded at a shear rate of 10 s^−1^ containing MC with the temperature was plotted. Two sections were identified on these plots. In the low temperature zone, the viscosity decreases with the raise of the temperature. This behavior was also observed in slurries dispersed with polyelectrolytes of both ceramic [30] and metallic powders [31]. As the temperature achieves the 50 °C, this decreasing in the viscosity is compensated and starts to increase as the MC already dissolved into the liquid medium starts to gel. The gelation process of MC occurs during heating due to the hydrophobic association between methyl groups on cellulose chains [32,33]. The points where derivate of the curves included in Figure 2b equals to 0 indicate the temperature where gelation process become predominant on the rheological behavior of the slurries. This temperature is 51, 49 and 47 °C, respectively, for the concentrations of MC of 8, 10 and 12 g/L. 

For the three concentrations when the temperature of the slurries overcome the 52 °C a significant increment in the viscosity was observed, as the gelation process become massive and the hydrophobic effective units starts to join the MC chains in a 3D structure [33]. Finally, at temperatures over 57 °C the viscosity increases exponentially, this temperature was reported as gelation temperature by Knarr and Bayer for cordierite slurries with MC [34]. All the previous results indicate that slurries will keep the shape of the container when they are heated at temperatures over 57 °C, due to the increment of viscosity induced by temperature. In addition to the shape, the material could keep the internal structure or microstructure that could be formed inside the slurry, such as gradient or porosity. These temperatures are coincident with the studies on dynamic viscoelasticity behavior with temperature for methylcelluloses solution which also reported that hysteresis curve finish at 70 °C [35].

**Figure 3:**
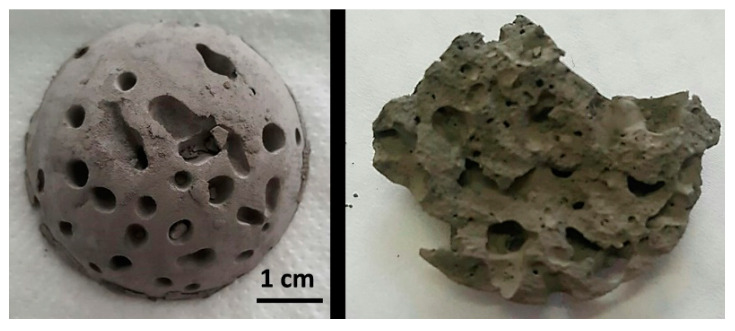
Porous Ti green sample, (**left**) dome shaped by gelation and drying and (**right**) a detail of the internal porous structure observed after breaking apart the sample.

Furthermore, the addition of the ammonium bicarbonate to the slurries formulation as a gas porogen by thermal decomposition produces the pores formation at the same time that temperature increases the viscosity, leading to stable pores inside the final sample. A picture of a dome shaped sample and a detail of the internal porous structure after gelation and drying are show in Figure 3 staging the proposed approach.

**Figure 4:**
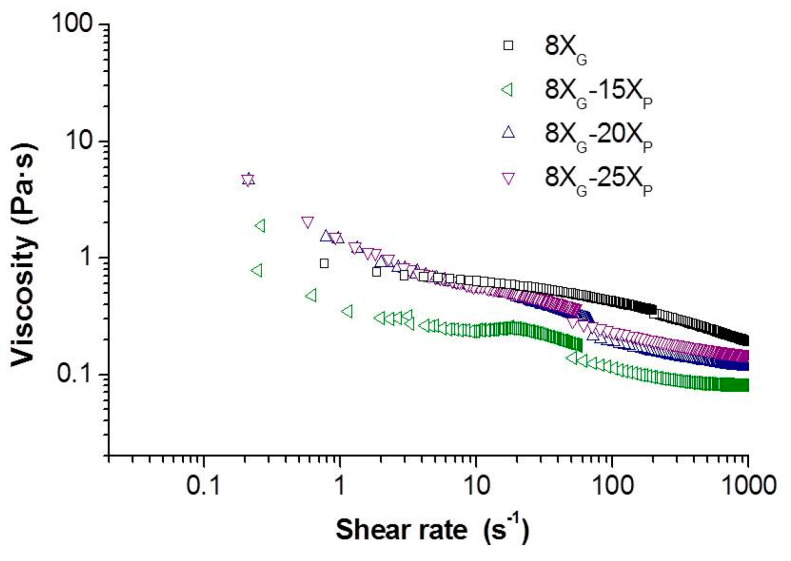
Evolution of the viscosity with the shear rate of 50 vol.% Ti slurries with 15, 20 and 25 wt.% of ammonium bicarbonate and 12 g/L of MC.

The flow curves of the slurries formulated dissolving different amounts of the MC (8, 10 and 12 g/L) and the porogen agent (15, 20 and 25 wt.% of ammonium bicarbonate related to the solid content) were determined. The plot in Figure 4 presents the rheograms of the viscosity evolution versus the shear rate for slurries with 12 g/L of MC and three contents of porogen (slurries (8X_G_−15X_P_), (8X_G_−20X_P_) and (8X_G_−25X_P_)). Similar rheograms for MC concentrations of 8 and 10 g/L were measured (no show here) with similar results and evolution of the slurries viscosity.

For a given amount of MC, the addition of 15 wt.% of ammonium bicarbonate leads to a decreasing in the viscosity of the slurry. This rheology sub serves the filling processed of the mold and is explained by the temperature dependence of a relatively big anions (bicarbonate anions) to the media [36,37]. Further additions of ammonium bicarbonate show an increment in the viscosity of the samples, as the increment of the ionic concentration led to a constriction on the electrical double layer and formation of short-range repulsive forces [38]. The rheological behavior of these slurries is plastic or pseudoplastic, similarly to the curves of the Figure 2, and they were adjusted to the Cross model to obtain the characteristic viscosity values of zero-shear viscosity (*η*_0_), infinite shear viscosity (*η*_∞_) and viscosity at 100 s^−1^ (*η*_100_).

**Figure 5:**
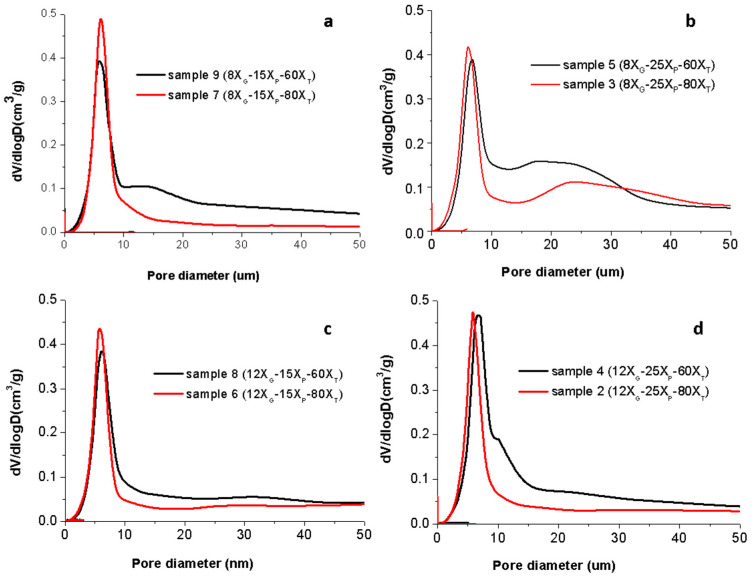
Pore distributions by Mercury Intrusion Porosimetry (MIP), for samples in Table S1. Grouping operational variables by fixing X_G_ and X_P_ and variable X_T_: (**a**) sample 9 (8X_G_−15X_P_−60X_T_) and sample 7 (8X_G_−15X_P_−80X_T_); (**b**) sample 5 (8X_G_−25X_P_−60X_T_) and sample 3 (8X_G_−25X_P_−80X_T_); (**c**) sample 8 (12X_G_−15X_P_−60X_T_) and sample 6 (12X_G_−15X_P_−80X_T_); (**d**) sample 4 (12X_G_−25X_P_−60X_T_) and sample 2 (12X_G_−25X_P_−80X_T_).

After demolding, the apparent density of dome bulk pieces was calculated and the pore size distribution was determined by MIP for the Ti green samples shaped under the 15 experimental conditions of the experimental design method (Table S1, supplementary material). Figure 5 shows the pore size distributions measured when the porogen concentration varies for 4 fixed conditions of MC concentration and temperature considered in the experimental design (corresponding to (a) samples 9 (8X_G_−15X_P_−60X_T_) and 7 (8X_G_−15X_P_−80X_T_), (b) samples 5 (8X_G_−25X_P_−60X_T_) and 3 (8X_G_−25X_P_−80X_T_), (c) samples 8 (12X_G_−15X_P_−60X_T_) and 6 (12X_G_−15X_P_−80X_T_), (d) samples 4 (12X_G_−25X_P_−60X_T_) and 2 (12X_G_−25X_P_−80X_T_)). Similar pore size distributions were determined for other samples in Table S1 (data and plots can be found as supplementary material) with similar results that those discussed below. 

The experimental values (also included as Supplementary Material, Table S2) of the formulated slurries, viscosities (*η*_0_, *η*_∞_ and *η*_100 s^−1^_), the apparent densities (D) and the mean pore diameter (P) resulting from the characterization of Ti sponges obtained at the 15 proposed experiments. Reported values show that viscosity in resting conditions varies from 1720 to 250 mPa·s and the mean pore size diameters range from 6.09 to 9.93 µm while the green density of Ti sponges varies from 1.77 to 2.56 g/cm^3^ (from 39.25% to 56.76%). 

To study the influence of each processing variable (X_G_, X_P_ and X_T_) in the apparent density, data in Table S2 and the plots in Figure 4 and similar (show as Supplementary Material, Figure S1) were analyzed. As a function of the ammonium bicarbonate concentration (when X_G_ and X_T_ were fixed varying the X_P_) and the MC concentration (X_P_ and X_T_ are fixed), whatever it is the values of other two variables, the apparent density decreases due to the higher amount of gas generated by the ammonium bicarbonate decomposition and the stronger gel structure formed by the slurry which is retaining the gas bubbles. In fact, from the microstructural inspection, pores in the green samples obtained with higher MC contents are lower in size and better distributed (Figure 3). However, the increment of the process temperature promotes the increase of the apparent density. This could be due to the faster gas formation when temperature increases that promotes the coalescence of the bubbles at low gel concentrations and high porogen contents, avoiding an effective formation of the gel. In those cases, even the full structure of the green sponge can collapse.

The influence of the variables in the pore sizes deriving from the data collected in Table S2 and the plots of pore distributions from the MIP such as those presented in Figure 5 and other similar with different X_P_ at the same X_T_ or X_G_. (not show here, but in the Supplementary Material, Figure S2) were also analyzed. As a function of the ammonium bicarbonate concentration (when X_G_ and X_T_ were fixed varying the X_P_), although the full width at half maximum for the main peak in the pore size distribution graphs ranges between 4 and 10 µm in all the cases, for the higher concentration of porogen a second broad area of porosity over the 10 µm is detected (Figure 5b,d). Similarly occurs if the temperature is considered as the variable (when X_G_ and X_P_ were fixed varying the X_T_), the higher is the temperature the wider is the population of pores with diameters higher than 10 µm. An in both cases, the higher is the concentration of the gelation agent (when X_P_ and X_T_ are fixed being X_G_ the variable) the lower is the extension of this second population of pores. Those results indicate that the gel strength prevents the coalescence of pores. However, for the highest gelation temperature tested, even the highest concentration of MC is not enough to keep the pores isolated, and then they coalesce to generate pores of bigger sizes.

The size of the pores is a critical parameter into the biological and mechanical response of the Ti based biomaterials. Furthermore, the porosity determines the mechanical response of the green sponges during handling. In order to evaluate the mechanical response of Ti green sponges, compression tests were performed on dome shaped samples at a displacement speed of 0.02 mm/s. In this test the applied compressive force distributes inside the sample transmitting the applied force in a simple elastic-plastic behavior [39]. The dome configuration makes the compressive forces to develop circular tensile stresses on the surface of the sample [40,41,42]. It has to be noted that this analysis considers a homogeneous and isotropic pore distribution without pore orientation, but orientation arising from the processing technologies can conditions the mechanical properties [43]. 

**Figure 6:**
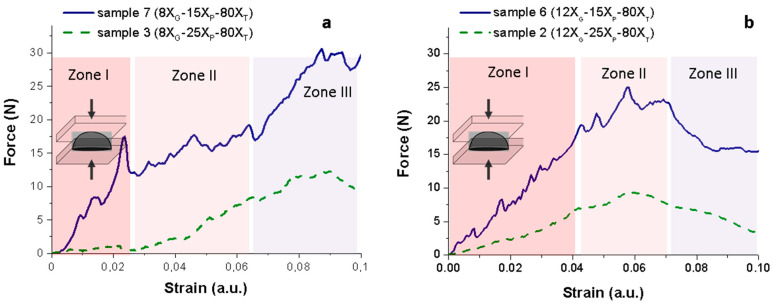
Force-strain curves recorded in the compression tests of green samples: (**a**) 7 (8X_G_−15X_P_−80X_T_) and 3 (8X_G_−25X_P_−80X_T_); (**b**) samples 6 (12X_G_−15X_P_−80X_T_) and 2 (12X_G_−25X_P_−80X_T_) in Table S1.

Plot in Figure 6 shows the force-strain curve recorded in these mechanical tests where three regions of deformation corresponding to different phenomena can be identified [44]. Initially the sponge response is nearly elastic (Zone I), where random tensile drops are due to the cracks initiated on the pores, i.e., properly are the structural defects and the rearrangement of the compacted powders. The strain value reached for zone I on Figure 6a,b is different because the higher content of gelling agent in 12 X_G_ samples provide a plastic binder between particles, what allows higher strain (and stress) without breaking. The second zone (Zone II) consists in a stress plateau where multiple cracks grow and propagate easily throughout the materials collapsing the structure. The transition from the Zone I to the Zone II define a yield point which depends on the size, connectivity and number of pores. The final evolution of the force-strain curve (Zone III) can be identified with a densification process forced by the uniaxial pressure that compression plates exert onto the broken sample. As the broken pieces are not confined into a die, the Zone III can’t be clearly identified [40]. In this study we were focused on the Zones I and II where the structural integrity of green sponges is mainly conditioned by the porosity and then by the selected operation variables (X_G_, X_P_ and X_T_). 

Plots of Figure 6a,b shows the load vs. deformation curves for the samples. In this case, 7 (8X_G_−15X_P_−80X_T_) and 3 (8X_G_−25X_P_−80X_T_), and samples. 6 (12X_G_−15X_P_−80X_T_) and 2 (12X_G_−25X_P_−80X_T_), respectively, which illustrate the mechanical response of Ti sponges obtained formulating slurries with 8 g/L and 12 g/L of MC, respectively, when the content of porogen change from 15 to 25 wt.% for a similar thermal treatment at 80 °C.

Results show that the porogen content strongly determines the structural integrity of the Ti sponges. In good agreement with the results described above, the curves force-strain exhibiting a higher slope correspond to the sponges obtained formulating the lower amount of ammonium bicarbonate (X_P_ = 15 wt.%), that means to the structures with a lower level of porosity. In fact, samples 7 (8X_G_−15X_P_−80X_T_) and 6 (12X_G_−15X_P_−80X_T_) have similar apparent densities (2.44 and 2.37 g/cm^3^, respectively) and mean pore sizes (6.22 and 6.62 µm, respectively). However, the comparative examination of the whole curve evolution of both samples suggests a structure reinforcement achieved by the incorporation of a higher amount of the MC. At the plots, the higher is the amount of CM in the slurry formulation the higher is the compressive force required to achieve the plateau at the Zone II. Moreover, the tensile drops are less abrupt in the sponge num. 6 (12X_G_−15X_P_−80X_T_). Those effects can be related to the joining effect of the gelation agent over the particles and that the porosity generated by the ammonium bicarbonate decomposition is clearly trapped by a stiffer and stronger gel structure, respectively.

On the other hand, samples 3 (8X_G_−25X_P_−80X_T_) and 2 (12X_G_−25X_P_−80X_T_) have dissimilar apparent densities (2.15 and 1.77 g/cm^3^, respectively) and mean pore sizes (7.17 and 6.27 µm, respectively). A low stiffness is observed for these sponges since they show a higher displacement for the application of smaller compressive forces. However, it is important to note that the whole behavior of the force-strain curves is similar to the curves measured for lower porogen contents (samples 7 and 6 Supplementary Material). So we can intuit that MC concentration influences the pore size and the porosity distribution, being both values higher as more porogen is added.

### 3.2. A Multiple Regression Analysis of the Experimental Data 

In order to evaluate the weight of the main variables of the process, into the final properties of the shaped materials, a multiple regression analysis of the experimental data collected in Table S2 was made with the software BMDP© to obtain a second-order polynomial equations relating each dependent variable (*η*_0_, D and P) with the operational variables (X_T_, X_G_ and X_P_), resulting in:

(5) $$D=2.2863+0.1493\cdot X_{P}^{2}-0.089\cdot X_{G}+0.105\cdot X_{T}-0.1290\cdot X_{P}-0.3307\cdot X_{G}^{2}$$

(6) $$P=7.1257-0.345\cdot X_{G}\cdot X_{P}-0.3970\cdot X_{G}+0.7557\cdot X_{P}^{2}-0.8093\cdot X_{T}^{2}-0.5170\cdot X_{T}+0.704\cdot X_{P}$$

(7) $$\eta_{0}=1.6946-0.2538\cdot X_{G}\cdot X_{P}-0.2350\cdot X_{G}+0.2983\cdot X_{P}-1.1396X_{G}^{2}$$

The values of apparent density, viscosity and mean pore size of the bulk pieces are satisfactorily adjusted to the equation of the polynomial model tested, with 5, 6 and 7, different terms that contain the independent variables (operation variables), their square values or binary combinations of the aforementioned variables, respectively. As observed in the previous equations, there are significant interactions between the three selected input variables (X_T_, X_G_ and X_P_) in each of the output variables (*η*_0_, D and P).

The polynomial models tested provide a good fit of the experimental data for the calculation of all response variables, as indicated by the values of multiple R, R2 and adjusted-R2 of the adjustments of Equations (5)–(7), as well as the higher *p*-values and the lower values of the Snedecor’s F parameter (for a 95% confidence interval). They are all summarized in Table 2.

The values estimated using the above Equations (5)–(7), reproduce the experimental results of the different dependent variables considered with errors less than of 10% in the most of cases. The following Table 3 shows the errors determined experimentally and the estimated data from the aforementioned equations. 

The analysis of the values collected in Table 3 shows that the worst estimation for the apparent density, D, with respect to the experimental values has an error of 12.53%, however this test corresponds to limit conditions of the proposed experiment, that is it corresponds to the maximum values of the three operation variables: 12 g/L of MC, 25 wt.% of porogen and 80 °C. Therefore, the adjustment made is more precise with medium-low values of the operation variables. The other estimated data reproduce the experimental values with errors lower than 7%, which is the lowest error of the three estimated variables. On the other hand, the calculated values of the zero-shear viscosity (*η*_0_) fits the experimental measured values with errors lower that 9%, the average value of the errors being lower than the rest of the variables, which indicates a good adjustment of the experimental results. Finally, the fitting values for the mean pore size (P) adjust to the experimental values with error lower than 15%. In this case, there is a greater disparity in the errors calculated on this variable from this model.

According to these results it can be concluded that calculated equations through a second order polynomic model for the processing of porous materials by a gelation technique conducts to error values lower than 7, 9 and 15%, in the worst case, for the apparent density, zero-shear viscosity and mean pore size, respectively. In this study, the errors assigned to the output variables are not significant within the range of the experimentally determined values, since the errors are within the range of variability and therefore the proposed model can be considered acceptable.

Those equations were used to determine the influence of the operational variables (input variables) on the properties of the slurries and the green structure of Ti bulk pieces (output variables). In this sense, the maximum and minimum values for each parameter (*η*_0_, D and P) were estimated from Equations (5)–(7), and the values of the corresponding operational variables (X_T_, X_G_ and X_P_) were determined, and collected in Table 4. 

Matrices (*x*; *y*; *z*) correspond to X_G_, X_P_ and X_T_, respectively. The viscosity values only depend on two variables, X_G_ and X_P_, as the temperature was applied after the determination of the slurry viscosity. To determine the influence of the input variables on the properties considered it is necessary to determine the optimum conditions for each of the mentioned properties. For that, a non-linear programming as implemented in the More and Toraldo method was used. The values of two of the input variables were fixed and the other one was varied until the maximum and minimum value of each output parameter was reached, and with them the error was calculated. The maximum error corresponds to the most influential operational variable on the output parameter (*η*_0_, D or P). 

To avoid particles segregation or sedimentation, the highest value of the zero-shear viscosity should be considered. For that reason, the optimum are the values which make maximize this parameter (−0.2; 1). As we previously said, the temperature value wasn’t a determinant factor, so the study is reduced only to two variables in which one or another is fixed, until achieving the maximum and minimum viscosity values. If X_G_ was fixed and X_P_ was the variable, the maximum and minimum viscosity values are 0.36 and 2.05, respectively, with error values of the 82.18%. Otherwise, when X_P_ was fixed and X_G_ was the variable, the values were 1.34 and 2.05 with and error of 34.38%. Consequently, the most influential variable in the zero-shear viscosity is X_P_.

In the other way, it has to be considered that the final objective was to achieve the lowest apparent density, which corresponds to the conditions for the highest porosity (1; 0.43; −1). Therefore, when X_G_ and X_P_ were fixed and X_T_ was varied, the maximum and minimum density values were 1.73 and 1.94 g/cm^3^ with an error of 10.80%. When X_G_ and X_T_ were fixed and X_P_ was varied, there are obtained minimum and maximum density values of 1.73 and 2.04 g/cm^3^ with an error of 15.00%. In addition, when X_P_ and X_T_ were fixed and X_G_ was varied, there were obtained minimum and maximum values of 1.73 and 2.16 g/cm^3^ with an error of 19.66%. So, the most influential variable in the density is X_G_, followed by the X_P_ and the lower influential was X_T_. 

Finally, for the porosity, it was desirable the higher values for the mean pores size, which resulted in the following conditions (−1; 1; −0.32). Fixing X_G_ and X_P_ and changing the X_T_, the maximum and minimum pore sizes were 9.4 and 8.0, respectively, with error of 14.92%. Fixing X_G_ and X_T_ and changing the X_P_, the maximum and minimum pore sizes were 9.4 and 7.2 µm, respectively, with an error value of 22.98%. In addition, fixing X_P_ and X_T_ and changing the X_G_, the maximum and minimum mean pore sizes were 9.4 and 7.93 µm, respectively, with an error value of 15.77%. Based on this data the most influential variable in the mean pore size is X_P_, followed by X_G_ and X_T_. 

Table 5 summarizes the results of the study about the estimation of the influence of operational variables in the processing parameters of the green Ti sponges.

### 3.3. Sintering and Characterization of Ti Sponges

**Figure 7:**
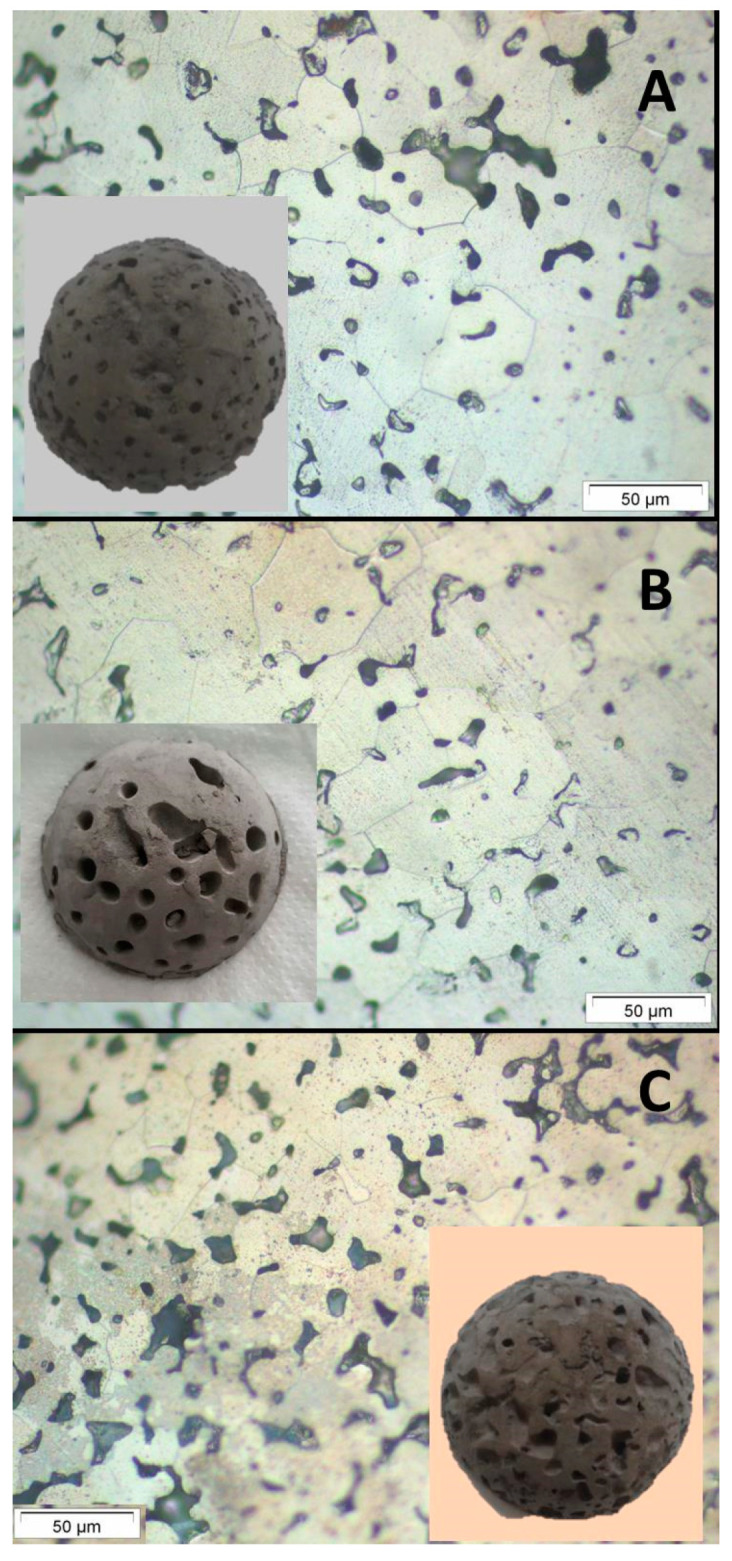
Pictures and micrographs of the porous titanium sintered bodies representing the different processing categories (**A**–**C**).

The porosity and the structure of the Ti structures after sintering were also studied. The Ti sponges were grouped in four categories: A, B, C and D. The Category A corresponds to the lower conditions for the operation variables (samples 8 and 9 in Table S1). These materials have the lower porosity. The low viscosity promotes the displacement of big pores (supra-macro porosity, P_supra-macro_) to the surface of the piece and burst lowering the porosity. Category B (samples 6, 7 and 11 in Table S1) is associated to the materials with bigger pores in which the pores can internally merge into big pores but has only a limited capability to move up to the surface and once they burst, do not lose the shape. This porosity was opened, tubular and directional to the surface. Category C (samples 1, 4, 13 and 14 in Table S1) corresponds to structures with a higher amount of pores, homogeneously distributed in the structure. In these cases, the viscosities were higher enough to keep the size of the pores without merges them in bigger ones but with open junctions. Finally, the Category D (samples 2, 3, 5, 10, 12 and 15 in Table S1) corresponds to the higher values of the operational variables. The processing of these slurries is complicated due to both, the high viscosity and porosity, promotes the structure collapse. Those materials are hardly handling and then category D has not been considered for further characterizations. Figure 7 shows the pictures and micrographs of the materials which represents the categories A, B and C, while Table 6 collects the characterization of the porosity and oxygen content (O_2_) of these materials after sintering. 

Materials have open porosities ranging from 19 to 39%, being the macro-porosity (P_macro_ ranging 50 nm < P < 200 µm) the more representative pore size in the Ti sponge microstructure. Samples on group A are denser than others and mainly present macroporosity. Materials on group B exhibit higher porosities which correspond mainly to the macro and supra-macro porosity. The high value on the supra-macro porosity was due to the coalescence of pores in the conditions where the gelation agent does not provide enough strength. Finally, materials of group C present the higher porosity, mainly macro porosity. The oxygen content ranges from 1.31 to 2.22 wt.%, close to the values of Ti processed by other aqueous colloidal techniques [19,22], being the structures on group C have the higher oxygen content, as a consequence of the higher total porosity.

It is important to note that samples of group B (6, 7 and 11 in Table S2) has the highest apparent green densities (2.37–2.50 g/cm^3^ corresponding to 51.40–54.23%) and a narrow pore size distribution with mean size ranging 6–7 µm. After sintering, these samples have the lowest closed porosity (0.2%) denoting the homogeneous and high degree of particles packing achieved during shaping. Moreover, these sponges exhibit the best compressive behavior (Figure 6) as a result of the homogeneous distribution of the generated porosity in the stiff green structure. Finally, the macro porosity in this category of sponges is balanced between macro- and supra-macro pores leading to sintered Ti sponges with 39% of open porosity and almost null close porosity.

## 4. Conclusions

Disperse and stable Ti aqueous slurries were formulated for the fabrication of porous materials by the incorporation of MC as a gelation agent and ammonium bicarbonate as a porogen. Ti sponges were then shaped by casting followed by a mild temperature treatment for gelation and drying. The thermal treatment promotes at the same time the gas formation gelation of the MC trapping the gas bubbles and leading to a stiff porous green structure. After sintering at 1100 °C under ultrahigh vacuum, Ti sponges with 39% of open porosity and almost null close porosity were obtained by adjusting the processing parameters. 

An experimental design analysis allows stablishing the conditions to adjust the porosity stablishing as processing variables the gelation agent and porogen concentration, and the temperature. Multiregression analyses result in the most influential variable in the zero-shear viscosity of the slurry and the mean pore size of packing is the porogen content, while the most influential variable in the apparent density is the concentration of the gelation agent in the slurry formulation.

Finally, the analysis of the experimental results as a function of the operating (processing) variables revels that the gel strength prevents the coalescence of pores reinforcing the structure of the green Ti sponge. The use of 12 g/L MC results in a well distributed and homogeneous porosity, while the highest level of porogen addition (25 wt.%) and the highest temperature (80 °C) produces the poorest mechanical stability of the green structure. The porosity of the green pieces obtained with higher MC contents is lower in size and better distributed resulting in a well consolidated Ti skeleton of the porous structure after sintering. 

## Figures and Tables

**Table 1:** Characteristic viscosity values for 50 vol.% Ti slurries formulated without MC and with 8, 10 and 12 g/L of MC.

Formulation	*η*_0_ (mPa·s)	*η*_∞_ (mPa·s)	*η*_10 s^−1^_ (mPa·s)	*η*_100 s^−1^_ (mPa·s)
without MC	430	10	410	61
8X_G_	760	95	620	396
10X_G_	900	108	735	458
12X_G_	1370	121	1070	515

**Table 2:** Statistical parameters values of polynomial models found for the processing of Ti porous structures.

Equation	Multiple-R	R^2^	Adjusted R^2^	*p* <	F >
D [Equation (3)]	0.86	0.73	0.59	0.16	2.34
P [Equation (4)]	0.85	0.73	0.52	0.24	1.61
*η*_0_ [Equation (5)]	0.99	0.99	0.99	1.12 × 10^−8^	381.20

**Table 3:** Statistical parameters values of polynomial models found for the processing of Ti porous structures.

Num.	D (g/cm^3^)	D (g/cm^3^) Estimated	Error (%)	*η*_0_ (Pa·s)	*η*_0_ (Pa·s) Estimated	Error (%)	P (μm)	P (μm) Estimated	Error (%)
1	2.38	2.29	3.78	1.72	1.69	1.48	7.33	7.13	2.79
2	1.77	1.99	12.53	0.35	0.36	4.15	6.13	6.53	6.32
3	2.15	2.17	0.92	1.34	1.34	0.15	7.11	8.00	12.53
4	1.85	1.78	3.68	0.35	0.36	4.15	7.24	7.55	4.30
5	1.98	1.96	1.02	1.35	1.34	0.59	9.28	9.04	2.64
6	2.37	2.25	5.07	0.28	0.28	1.62	6.20	5.80	4.93
7	2.44	2.43	0.50	0.26	0.24	8.48	6.27	5.90	5.85
8	1.95	2.04	4.61	0.28	0.28	1.62	6.98	6.83	2.10
9	2.24	2.22	0.99	0.26	0.24	8.48	7.07	6.94	1.88
10	2.46	2.31	6.24	-	-	-	9.93	8.59	13.54
11	2.50	2.56	2.58	1.32	1.40	5.78	6.23	7.18	15.21
12	2.56	2.39	6.59	1.72	1.69	1.48	6.41	5.80	9.53
13	2.22	2.18	1.74	1.72	1.69	1.48	6.62	6.83	3.22
14	1.99	1.87	6.20	0.34	0.32	5.88	6.48	6.73	3.84
15	2.01	2.04	1.72	0.74	0.79	6.76	7.17	7.52	4.92

**Table 4:** Operational variables values in the processing of Ti sponges to obtain maximum and minimum values of the dependent variables.

Parameter	Maximum Values		Minimum Values	
	(X_G_; X_P_; X_T_)	Results	(X_G_; X_P_; X_T_)	Results
D (g/cm^3^)	(−0.13; −1; 1)	2.67	(1; 0.43; −1)	1.74
*η*_0_ (Pa·s)	(−0.2; 1)	2.04	(−1; −1)	0.23
P (μm)	(−1; 1; −0.32)	9.41	(1; −0.24; 1)	5.35

**Table 5:** Summary of the estimation of the influence of operational variables in the processing parameters of Ti sponges.

Parameter	Operational Variable	Errors	Influence
Apparent Density (D)	Fixed: X_G_, X_P_ Variable: X_T_	10.80%	X_G_ > X_P_ > X_T_
	Fixed: X_G_, X_T_ Variable: X_P_	15.00%	
	Fixed: X_P_, X_T_ Variable: X_G_	19.66%	
Zero-shear Viscosity (η_o_)	Fixed: X_G_ Variable: X_P_	82.18%	X_P_ > X_G_
	Fixed: X_P_ Variable: X_G_	34.38%	
Mean Porous Size (P)	Fixed: X_G_, X_P_ Variable: X_T_	14.92%	X_P_ > X_G_ > X_T_
	Fixed: X_G_, X_T_ Variable: X_P_	22.98%	
	Fixed: X_P_, X_T_ Variable: X_G_	15.77%	

**Table 6:** Porosity characterization of the sintered Ti sponges including their associated oxygen content.

Category	P_Total_ *	P_closed_	P_open_	P_micro-meso_ (<50 nm)	P_macro_ (<200 µm)	P_supra-macro_ (>200 µm)	O_2_ (wt.%)
A	20.4%	1.7%	18.7%	<0.005%	18.3%	0.4%	1.54%
B	39.4%	0.2%	39.2%	<0.005%	20.3%	18.9%	1.31%
C	42.2%	7.8%	34.4%	<0.005%	33.1%	1.3%	2.22%

* Determined by geometric calculations and considering a theoretical density of 4.61 m/cm^3^.

## Data Availability

Data sharing is not applicable to this article.

## Supplementary Materials

The following are available online at https://www.mdpi.com/article/10.3390/ma14164744/s1, Figure S1: Evolution of the viscosity with the shear rate of suspensions with 15, 20 and 25% wt. BA and (a) 8 g/L of MC, (b)10 g/L of MC and (c) 12 g/L of MC. Figure S2: Mercury Instrusion Porosimetry Curves of the samples with different XP at the same XT or XG, Table S1: Normalized values of the operational processing variables, Table S2: Summary of experimental values corresponding to the viscosities, apparent densities and mean pore diameters of samples obtained from experimental design defined in Table S1.
