# Supplementary material for: Operational Variables on the Processing of Porous Titanium Bodies by Gelation of Slurries with an Expansive Porogen
Source: Materials (Basel). 2021 Aug 22;14(16):4744. doi: 10.3390/ma14164744 (PMC8399247; doi:10.3390/ma14164744)
Supplement: Supplementary file 1 [file materials-14-04744-s001.zip › materials-1284352-supplementary.pdf]

Supplementary Materials

Table S1. Normalized values of the operational processing variables.

Num.	Experiment	X_G (g/L)	X_P (%)	X_T (°C)
1	**0 0 0**	10	20	70
2	**1 1 1**	12	25	80
3	**-1 1 1**	8	25	80
4	**1 1 -1**	12	25	60
5	**-1 1 -1**	8	25	60
6	**1 -1 1**	12	15	80
7	**-1 -1 1**	8	15	80
8	**1 -1 -1**	12	15	60
9	**-1 -1 -1**	8	15	60
10	**0 1 0**	10	25	70
11	**0 -1 0**	10	15	70
12	**0 0 1**	10	20	80
13	**0 0 -1**	10	20	60
14	**1 0 0**	12	20	70
15	**-1 0 0**	8	20	70

Table S2. Summary of experimental values corresponding to the viscosities, apparent densities and mean pore diameters of samples obtained from experimental design defined in table S1.

Num.	X_G (g/L)	X_P (%)	X_T (°C)	η_0 (mPa·s)	η_∞ (mPa·s)	$\eta_{100s^{-1}}$ (mPa·s)	D (g/cm³)	P (μm)
1	0	0	0	1720	355	179	2.38	7.07
2	1	1	1	350	191	205	1.77	6.27
3	-1	1	1	1340	194	233	2.15	7.17
4	1	1	-1	350	191	205	1.85	9.28
5	-1	1	-1	1340	194	233	1.98	7.11
6	1	-1	1	280	67	153	2.37	6.22
7	-1	-1	1	250	165	143	2.44	6.62
8	1	-1	-1	280	67	153	1.95	7.33
9	-1	-1	-1	250	165	143	2.24	6.41
10	0	1	0	-	539	646	2.46	9.93
11	0	-1	0	1320	344	166	2.50	6.98
12	0	0	1	1720	355	179	2.56	6.09
13	0	0	-1	1720	355	179	2.22	6.49
14	1	0	0	340	127	187	1.99	7.24
15	-1	0	0	740	183	190	2.01	6.13

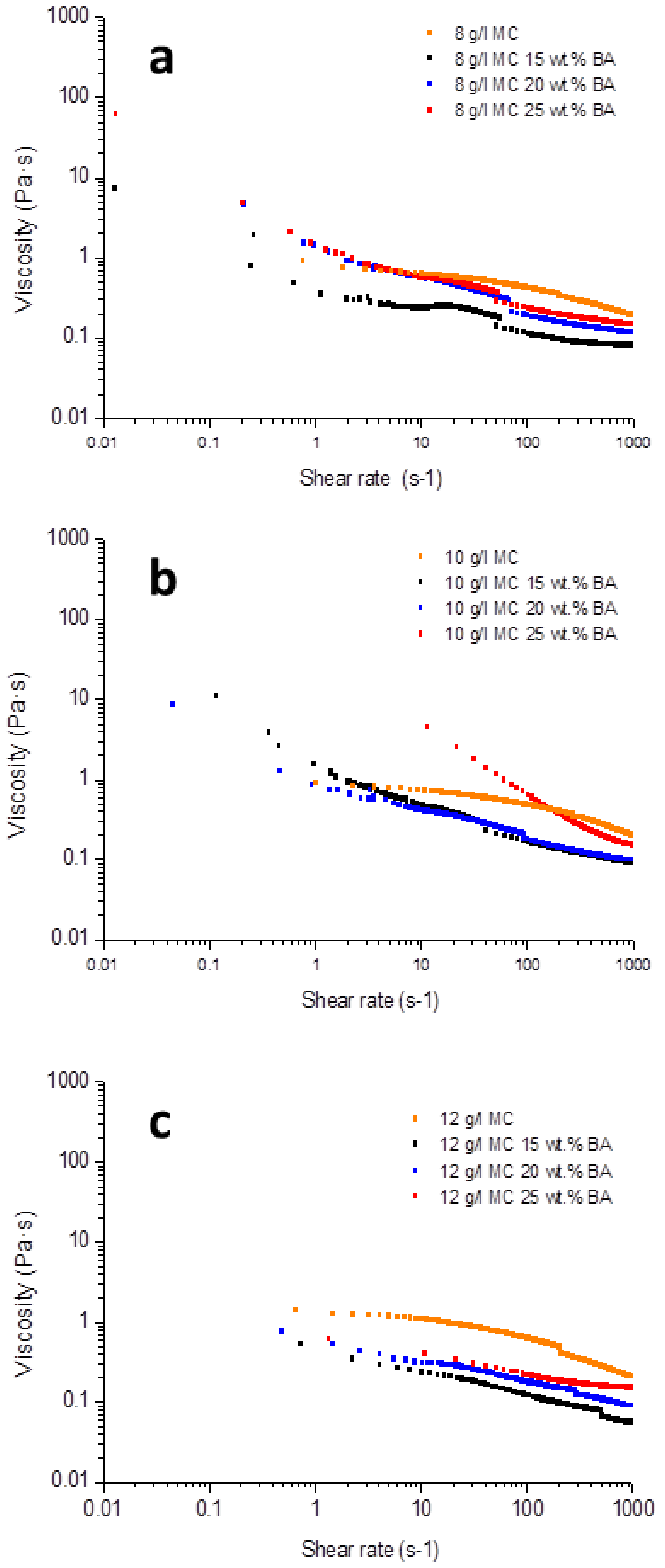

Figure S1. Evolution of the viscosity with the shear rate of suspensions with 15,20 and 25%wt. BA and a) 8g/L of MC, b)10g/L of MC and c) 12g/L of MC.

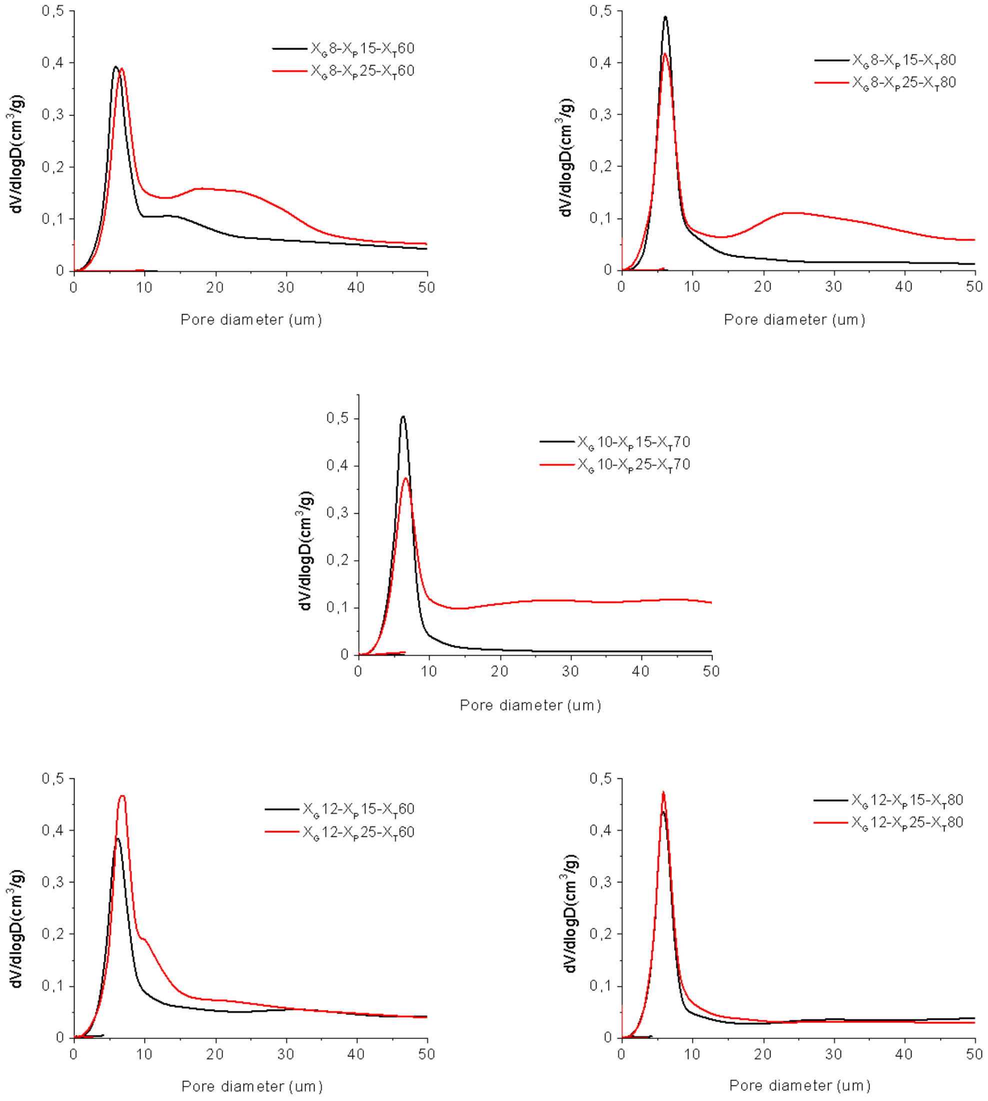

Figure S2. Mercury Instrusion Porosimetry Curves of the samples with different XP at the same XT or XG.
